# The relative efficacy of imatinib, dasatinib and nilotinib for newly diagnosed chronic myeloid leukemia: a systematic review and network meta-analysis

**DOI:** 10.1186/2162-3619-2-5

**Published:** 2013-02-19

**Authors:** Stuart Mealing, Leticia Barcena, Neil Hawkins, James Clark, Victoria Eaton, Ishan Hirji, Catherine Davis

**Affiliations:** 1 Oxford Outcomes Ltd Seacourt Tower, West Way, Oxford, UK; 2 Bristol-Myers Squibb, 5 Research Parkway, Wallingford, CT, 06492, USA; 3 Bristol-Myers Squibb, 100 Nassau Park Boulevard, Princeton, NJ, 08540, USA

**Keywords:** Dasatinib, CML, Chronic myeloid leukaemia, Network meta-analysis, Systematic review, Relative efficacy

## Abstract

**Objectives:**

Dasatinib 100 mg daily and nilotinib 600/800 mg daily have been compared to imatinib as first line treatments for CML in two recent randomised studies. However, no head to head evidence exists of the relative efficacy of dasatinib and nilotinib.

**Methods:**

We conducted a systematic literature review and used the data extracted to perform an indirect comparison meta-analysis of the three interventions.

**Results:**

Data from eight clinical studies (3,520 individuals) were included, all of which were of good quality (low risk of bias). At six months, the odds of complete cytogenetic response (CCyR) for dasatinib and nilotinib were approximately three times those for imatinib (range 2.77 to 3.06, all values not significant). At twelve months datatinib and nilotinib were significantly better than imatinib for both CCyR and major molecular response (MMR) (CCyR odds range 2.06 to 2.41, MMR odds range 2.09 to 2.87). At eighteen months dasatinib and nilotinib were again significantly better in terms of CCyR than imatinib (response odds 1.55 to 2.01). When dasatinib and nilotinib were compared to each other, for both clinical endpoints at all time points the response odds were not significantly different.

**Conclusions:**

On the basis of a systematic review of the current literature base, dasatinib 100 mg, nilotinib 600 mg and nilotinib 800 mg should be viewed as equivalent in terms of complete cytogenetic and major molecular response.

## Introduction

Chronic myeloid leukemia (CML) is a leukemia characterised by the t(9:22) translocation known as the Philadelphia chromosome. The annual incidence rate of CML is 1–2 per 100,000 people [1]. The majority of diagnoses are made in the chronic disease stage (CP-CML) as opposed to the accelerated or blastic stages [2,3].

Historically, while an allogeneic stem cell transplant offered the greatest chance of long term survival, individuals ineligible for transplant would be offered interferon-alfa (IFN-α), hydroxyurea or chemotherapy as first line therapy. Due to the largely palliative nature of these interventions – in particular hydroxyurea – the prognosis for patients receiving these treatments is poor, with expected survival of four to six years [2,3].

European LeukemiaNet and NCCN guidelines however, recommend that newly diagnosed individuals commence therapy with imatinib (Gleevec™, Novartis), an inhibitor of the oncogenic BCR-ABL protein present in CML, at a dose of 400 mg per day [4,5]. The efficacy of this product compared to interferon usage was demonstrated in the International Randomised Study of Interferon and STI571 (IRIS) [6]. Long term follow up studies from IRIS have highlighted the long term benefits of imatinib therapy [7-9]. Statistical modeling techniques have been used to extrapolate the data from IRIS, resulting in predicted mean survival estimates of approximately 20 years in patients who respond to treatment [10,11].

Recently, two second generation tyrosine kinase inhibitors (TKIs) have been developed (dasatinib, Bristol-Myers Squibb and nilotinib, Novartis) and represent viable alternatives to imatinib. These products were initially launched for use as second line therapies and were approved for first line use by the US Food and Drug Administration and the European Medicines Agency in 2010 on the basis of the results from two ongoing multinational RCTs [12,13].

In the Dasatinib versus Imatinib Study in Treatment Naïve CML Patients (DASISION) clinical trial [13], 519 individuals were randomised (1:1) to receive either dasatinib 100 mg or imatinib 400 mg daily. At 12 months, dasatinib was statistically superior in terms of both major molecular response (MMR, p = 0.007) and complete cytogenetic response (CCyR) (p < 0.001). Nilotinib 600 mg and 800 mg daily were compared to imatinib 400 mg daily in the Evaluating Nilotinib Efficacy and Safety (ENESTnd) clinical trial [12]. Randomisation was 1:1:1 and nilotinib was statistically superior at one year on the same endpoints (p < 0.001 for all comparisons).

Despite the demonstrable link between response to treatment and survival [8,14-16] agencies such as the United Kingdom’s National Institute for Health and Clinical Excellence (NICE) and the Canadian Agency for Drugs and Technologies in Health (CADTH) as well as private entities such as the Academy of Managed Care Pharmacy (AMCP) in the United States use both clinical and economic information in their assessment process [17-19]. In particular, estimates of relative efficacy are required in order to assess whether or not the additional benefits of a particular drug compared to another outweigh the additional costs. In this context, ‘benefit’ can refer to life years gained, Quality-adjusted life years gained (QALYs) or any other clinical endpoint.

At the time of manuscript preparation, there are no published randomised or observational head to head trials of dasatinib and nilotinib in newly diagnosed CML to inform decision making at either the payer (national) or prescriber (local) level. Further, any reimbursement decisions relating to the treatment of newly diagnosed CML will inevitably focus on the relative efficacy of both interventions compared to current standard treatment (imatinib 400 mg) as well as compared to each other.

We therefore undertook a systematic review of the literature on first line treatment to identify all randomised controlled trials of first line dasatinib, nilotinib and imatinib, and used the results from this review to generate relative efficacy estimates for key measures of treatment efficacy (CCyR and MMR) at as many time points as possible, using an indirect comparison meta-analysis. The results from this analysis will help all stakeholders overcome the lack of head to head data in making either reimbursement or prescribing decisions.

## Materials and methods

### Evidence identification

The Medline, Medline in Process and EMBASE databases were searched through the OVID SP portal. Database search strategies were designed using MeSH and Emtree terms respectively relating to CML, the treatments of interest and a randomised controlled trial filter. Restrictions were only made on studies published from 1980, publication language (English only) and population (adults greater than 18 years old). The search terms are reproduced in full in Additional file 1. In order to ensure the evidence network was as comprehensive as possible, relevant literature search terms were included for other interventions (i.e. interferon, hydroxyurea).

Conference proceedings from the 2009 and 2010 American Society of Hematology (ASH) and American Society of Clinical Oncology (ASCO) and the 2009 European Hematology Association (EHA) annual meetings were searched. In order to be as inclusive as possible, the project sponsors (Bristol-Myers Squibb, BMS) were invited to provide any as yet unpublished material for potential inclusion into the review.

In addition to type of publication and treatment option, the inclusion criteria used covered the need to report information on one of the endpoints of interest (CCyR, MMR) at any time point. Reasons for exclusion were non-randomisation, non-comparativeness, pharmacokinetic studies, animal/in vitro studies or publications without an available abstract.

Abstracts and sponsor-provided literature were independently screened by two reviewers against the formal inclusion criteria. Full papers were screened by two reviewers against the same inclusion/exclusion criteria used in the initial screening, with a third reviewer providing a casting vote where no consensus occurred. Data was then extracted into an Excel® workbook by one reviewer and checked by another.

The quality of all included articles was assessed using a series of bespoke questions derived from the *Cochrane Handbook for Systematic Review of Interventions*[20]. A description of the method used for the quality assessment is included in Additional file 1.

### Evidence synthesis

Indirect comparison (network) meta-analysis is an extension of conventional, pair-wise meta-analysis [21-25]. A statistical analysis of the network of trial evidence is used to produce comparable estimates of the effectiveness for a range of treatments. Both direct (i.e. head to head) and indirect evidence is used in this approach to evidence synthesis. Network meta-analysis is based on the assumption that, on a suitable scale, the difference in effect between treatments A & B is equal to the difference in effects between treatments A & C and B & C. The analysis can be expanded to more complex networks of evidence through the inclusion of additional interventions.

Studies have shown that the results from a network meta-analysis are consistent with those from a conventional meta-analysis [26] and, by their need for an assumed commonality of treatment effect modifiers across all included studies, respect randomisation in each individual trial. Of note, is that implicit in conventional meta-analyses is the assumption of either a common treatment effect or study-specific treatment effects distributed around a typical value [26].

A fixed-effects approach (i.e. common treatment effect) was used to estimate the probability of response at each time point. Given that it is the current standard of care, imatinib 400 mg daily was the baseline comparator against which all relative efficacy estimates were calculated. All calculations were performed on the log-odds scale, and hence results are expressed in terms of odds and absolute probability estimates.

The network meta-analysis was implemented in the WinBuGS and R software packages [27,28], with two Markov Chain Monte-Carlo chains used starting from different initial values of select unknown parameters. Each chain contained 20,000 burn-in iterations followed by 100,000 update iterations. Convergence was assessed by visualising the histories of the chains against the iteration number, since overlapping histories that appear to mix with each other provide an indication of convergence.

Since this approach is inherently Bayesian, initial values (‘priors’) are required to initiate the analysis, and we used non-informative Normal functions in order to have the results solely reflect the data used in the analysis, and not any *a priori* belief in the results. Inferences on the relevant parameters are based on the convergence of the derived posterior distributions. The accuracy of the posterior estimates was assessed via the calculation of the Monte Carlo error for each parameter.

## Results

### Total number of studies identified

The initial searches were performed on 9^th^ November 2009 and were updated on 18^th^ June 2010 and 31^st^ March 2011. After the elimination of duplicates, the EMBASE and MEDLINE searches identified a total of 2,024 abstracts of potential interest. The conference searches identified a further 534 potentially relevant publications. BMS made seven further articles and abstracts available. Following formal review, information reported in a total of 16 articles that relate to eight distinct clinical studies were also included in the analysis [12,13,29-41]. A summary of all included studies is presented in Table 1. Information derived from 3,520 individuals was used to inform the NMA.

**Table 1:** Description of included studies

**Study**	**Study characteristics**							**Assessment of study quality**					
	**Study design**	**Study duration (months)**	**Interventions**	**N**	**Median follow up (months)**	**Median dose**	**% lost to follow up**	**Q1**	**Q2**	**Q3**	**Q4**	**Q5**	**Q6**
DASISION [13,29-31]	Multicentre, Randomised, Phase III		Dasatinib 100 mg^a^	259	18.0	99 mg	15%	Yes	No	Yes	No	Yes	Yes
			Imatinib 400 mg^a^	260		400 mg	19%						
Baccarani et al. [32]	Prospective, randomised	12	Imatinib 400 mg^a^	108	26	400 mg		No	Yes	No	Yes	Yes	No
			Imatinib 800 mg^a^	108	26	720 mg							
German CML Study IV [33,34]	Randomised	36	Imatinib 800 g^a^	338	40	646 mg		Yes	No	No	No	No	No
			Imatinib 400 g^a^	326	40	400 mg							
ENESTed [12,35-37]	Multicentre, Randomised, Phase III	60	Nilotinib 300 mg^b^	282	18	592 mg	16%	Yes	No	Yes	No	Yes	Yes
			Nilotinib 400 mg^b^	281	18	779 mg	18%						
			Imatinib 400 mg^a^	283	18	400 mg	21%						
S0325 Intergroup [38]	Open Label, Randomised, Phase II	12	Imatinib 400 mg^a^	127				Yes	No	No	No	No	No
			Dasatinib 100 mg^a^	126									
ISTAHIT [39]	Multicentre, Randomised, Phase III	24	Imatinib 400 mg^a^	114	12.75	400 mg		Yes	No	Yes	No	Yes	Yes
			Imatinib 800 mg^a^	113	12.75	767 mg							
Cortes et al. [40]	Multicentre, Randomised, Phase III	12	Imatinib 400 mg^a^	157	17		0%	No	No	Yes	No	Yes	Yes
			Imatinib 400 mg^a^	319	17		0.6%						
SPIRIT [41]	Prospective, Randomised, Phase III	24	Imatinib 400 mg^a^	159	47	400 mg	0%	Yes	No	Yes	No	Yes	Yes
			Imatinib 600 mg^a^	160	47	590 mg	0%						

a) SID; b) BID; Q1) Was the method used to randomize patients described and adequate?; Q2) Was the method used for allocation concealment described and adequate?; Q3) Were the detailed inclusion/exclusion criteria described?; Q4) Was the method used to ensure treatment blinding described and adequate?; Q5) Were the patient baseline characteristics described? Q6) Were all analyses carried out using data from the Intention To Treat (ITT) patient group?

Of the abstracts and articles initially made available by BMS, two were dasatinib related manuscripts that were in the peer-review process and one was a separate manuscript in the development stage. In addition, three were abstracts subsequently presented at clinical conferences. All of these are now in the public domain and hence were identified during the search updates [29,31,38]. The final article related to the use of nilotinib in a non-randomised cohort of individuals and was thus excluded from the meta-analysis.

Five of the included studies compared imatinib at standard (400 mg daily) and high dose (600 mg/800 mg daily) [32-34,39-41] and these were included in the evidence network for reasons of completeness. Two studies compared dasatinib 100 mg daily to imatinib 400 mg daily [13,29-31,38] and one compared nilotinb 600 mg/800 mg daily to imatinib 400 mg daily [12,35-37].

**Figure 1:**
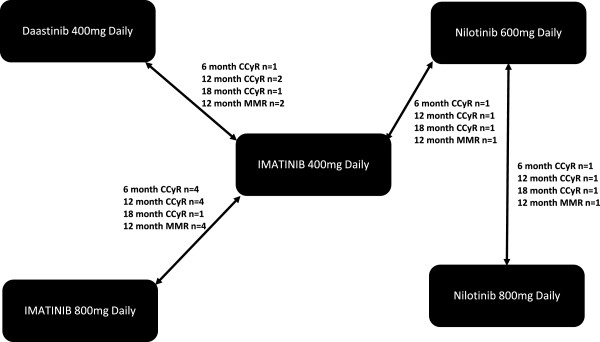
Evidence network diagram.

Meaningful evidence networks were created for CCyR at six, 12 and 18 months and for MMR at 12 months only (Figure 1). Information on CCyR was reported in seven studies at six and 12 months and in four studies at 18 months, and data on MMR at 12 months in seven studies (Table 2). With the exception of a general lack of reporting of methods used for blinding and concealment, the included studies are of good quality (i.e. low risk of bias) (Table 1).

**Table 2:** Data used in network meta-analysis

**Study**	**Dasatinib**	**Nilotinib 600 mg**	**Nilotinib 800 mg**	**Imatinib 400 mg**	**Imatinib 800 mg**
***Complete cytogenic response***					
**6 months**					
DASISION	73.0%			59.2%	
ENESTed		67.0%	63.0%	44.9%	
Baccarani et al.				50.0%	51.9%
German CML Study IV				21.5%	33.5%
Cortes et al. 2010				44.6%	56.7%
ISTAHIT				19.8%	43.8%
SPIRIT				50.0%	69.0%
**12 months**					
DASISION study group	83.4%			71.5%	
ENESTed study group		80.1%	77.9%	65.0%	
Baccarani et al. (2009)				58.3%	63.9%
German CML Study IV				49.8%	63.2%
Cortes et al. 2010				65.6%	69.9%
S0325 Intergroup Trial	82.0%			69.0%	
SPIRIT				58.0%	65.0%
**18 months**					
Dasatinib Study Group	88.6%				
DASISION study group	78.0%			70.0%	
ENESTed study group		85.0%	82.0%	74.0%	
German CML Study IV				66.6%	73.8%
***Major Molecular response***					
**12 months**					
Bacarrani et al. (2009)				33.3%	39.8%
DASISION study group	45.9%			28.1%	
ENESTed Study group		44.0%	43.1%	21.9%	
German CML Study IV				30.8%	54.8%
Cortes et al. 2010				40.1%	46.4%
SPIRIT				38.0%	49.0%
S0325 Intergroup Trial	59.0%			43.0%	

### Complete cytogenetic response (compared to Imatinib)

At both six and 12 months, the reported CCyR probabilities for imatinib in the DASISION study were higher than in all other observed trials.

At six months the odds of response for both dasatinib and nilotinib was approximately three times higher than for imatinib 400 mg daily, although these results were not significant (dasatinib 100 mg 2.98 [95% CrI 0.45 to 9.76], nilotinib 600 mg 3.06 [95% CrI 0.42 to 9.85], nilotinib 800 mg 2.77 [95% CrI 0.38 to 8.82]; Table 3). In addition, the derived probabilities show a high level of uncertainty, which arose due to the weak evidence network (dasatinib 100 mg 55.3% [95% CrI 22.9% to 86.7%], nilotinib 600 mg 60.7% [95% CrI 21.9% to 86.8%], nilotinib 800 mg 56.8% [95% CrI 20.4% to 85.5%]). However, by 12 months more robust evidence networks were created and as a result of the associated narrowing of all credible intervals the probability of response compared to imatinib for both products is significant (both intervals exclude one). The pooled one year CCyR estimates for both dasatinib and nilotinb were very similar (dasatinib 100 mg 77.1% [95% CrI 67.2% to 85.3%], nilotinib 600 mg 77.7% [95% CrI 64.8% to 87.7%], nilotinib 800 mg 75.3% [95% CrI 61.0% to 86.1%]). The 18 month estimates for dasatinib 100 mg and nilotinib 800 mg were again similar and significantly different to imatinib 400 mg (dasatinib 100 mg 79.1% [95% CrI 72.0% to 85.1%], nilotinib 600 mg 83.1% [95% CrI 76.7% to 88.4%], nilotinib 800 mg 80.0% [95% CrI 73.0% to 85.8%]).

**Table 3:** Complete cytogenetic response at 6, 12 and 18 months

**Intervention**	**Probability of response**	**Response odds ratios**
	**(Mean, 95%CrI)**	**(Mean, 95%CrI)**
***CCyR at 6 months***		
Imatinib 400 mg daily	40.1% (Reference Category)	N/A (Reference Category)
Dasatinib 100 mg daily	55.3% (22.9-86.7%)	2.98 (0.45-9.76)
Nilotinib 300 mg twice daily	60.7% (21.9-86.8%)	3.06 (0.42-9.85)
Nilotinib 400 mg twice daily	56.8% (20.4-85.5%)	2.77 (0.38-8.82)
Imatinib 800 mg daily	53.8% (35.7-71.8%)	1.92 (0.83-3.81)
***CCyR at 12 months***		
Imatinib 400 mg daily	62.4% (Reference Category)	N/A (Reference Category)
Dasatinib 100 mg daily	77.1% (67.2-85.3%)	2.16 (1.23-3.5)
Nilotinib 300 mg twice daily	77.7% (64.8-87.7%)	2.41 (1.11-4.29)
Nilotinib 400 mg twice daily	75.3% (61.0-86.1%)	2.06 (0.95-3.73)
Imatinib 800 mg daily	70.1% (62.1-76.9%)	1.45 (0.99-2.01)
***CCyR at 18 months***		
Imatinib 400 mg daily	71.6% (Reference Category)	N/A (Reference Category)
Dasatinib 100 mg daily	79.1% (72.0-85.1%)	1.55 (1.02-2.27)
Nilotinib 300 mg twice daily	83.1% (76.7-88.4%)	2.01 (1.31-3.00)
Nilotinib 400 mg twice daily	80.0% (73.0-85.8%)	1.63 (1.07-2.40)
Imatinib 800 mg daily	77.9% (71.9-83.2%)	1.43 (1.01-1.96)

### Major molecular response (compared to Imatinib)

At 12 months the odds of response for both dasatinib and nilotinib were at least two times higher than for imatinib 400 mg, with the results being significant (dasatinib 100 mg 2.09, nilotinib 600 mg 2.87, nilotinib 800 mg 2.76; Table 4).

**Table 4:** Major molecular response at 12 months

**Intervention**	**Mean**	**95%LCrI**	**95%UCrI**
***Probability of response***			
Imatinib 400 mg daily	33.6%	Reference category	
Dasatinib 100 mg daily	51.1%	43.9%	58.5%
Nilotinib 300 mg twice daily	58.7%	49.6%	67.5%
Nilotinib 400 mg twice daily	57.8%	48.7%	66.8%
Imatinib 800 mg daily	48.1%	43.1%	53.1%
***Response Odds Ratios***			
Imatinib 400 mg daily	N/A	Reference category	
Dasatinib 100 mg daily	2.09	1.55	2.78
Nilotinib 300 mg twice daily	2.87	1.95	4.11
Nilotinib 400 mg twice daily	2.76	1.88	3.98
Imatinib 800 mg daily	1.84	1.50	2.24

*LCrI*: Lower boundary of derived credible interval; *UCrI*: Upper foundry of derived credible interval.

The one year MMR probabilities for dasatinib and nilotinib were again shown to be significantly different to imatinib 400 mg (Table 4) and similar to each other (dasatinib 100 mg 51.1% [95% CrI 43.9% to 58.5%], nilotinib 600 mg 58.7% [95% CrI 49.6% to 67.5%], nilotinib 800 mg 57.8% [95% CrI 48.7% to 66.8%]).

### Head to head comparison (dasatinib and nilotinib)

A summary of the odds ratios generated for all endpoints when dasatinib was compared to nilotinib is presented in Table 5. Regardless of nilotinib dose, timepoint or endpoint of interest, all intervals include one, and therefore it is not possible to differentiate the two products in terms of either cytogenetic or major molecular response.

**Table 5:** **Head to head relative treatment effects expressed as odds ratios (mean, 95**% **CrI)**

	**Dasatinib 100 mg**	**Nilotinib 600 mg**	**Nilotinib 800 mg**
***CCyR (6 months)***			
Dasatinib 100 mg	N/A	0.76 (0.1, 9.31)	0.91 (0.12, 10.58)
Nilotinib 600 mg	1.32 (0.11, 9.58)	N/A	1.2 (0.23, 5.5)
Nilotinib 800 mg	1.10 (0.09, 8.29)	0.84 (0.18, 4.27)	N/A
***CCyR (12 months)***			
Dasatinib 100 mg	N/A	0.95 (0.41, 2.22)	1.09 (0.47, 2.60)
Nilotinib 600 mg	1.05 (0.45, 2.43)	N/A	1.15 (0.58, 2.32)
Nilotinib 800 mg	0.92 (0.38, 2.13)	0.87 (0.43, 1.73)	N/A
***CCyR (18 months)***			
Dasatinib 100 mg	N/A	0.77 (0.44, 1.36)	0.95 (0.54, 1.66)
Nilotinib 600 mg	1.30 (0.73, 2.29)	N/A	1.23 (0.79, 1.92)
Nilotinib 800 mg	1.05 (0.60, 1.84)	0.81 (0.52, 1.27)	N/A
***MMolR (12 months)***			
Dasatinib 100 mg	N/A	0.74 (0.45, 1.19)	0.76 (0.47, 1.22)
Nilotinib 600 mg	1.35 (0.84, 2.20)	N/A	1.04 (0.74, 1.45)
Nilotinib 800 mg	1.31 (0.82, 2.13)	0.96 (0.69, 1.35)	N/A

## Discussion

The short term clinical efficacy of imatinib, dasatinib and nilotinib has been demonstrated in large, well designed randomised trials [6,12,13]. Long term data from the IRIS clinical trial is strongly supportive of the argument that individuals who receive first line imatinib will live many years longer than those who receive interferon based therapy [7,9]. Due to the causal link between prognosis and response to treatment it is highly likely, given the significant differences observed in complete cytogenetic and major molecular response rates for second generation compared to first generation TKIs, that a similar pattern will emerge when long term data from DASISION and ENESTnd are published.

Despite the positive clinical profile of dasatinib and nilotinib in the treatment of newly diagnosed CML, reimbursement agencies such as NICE and CADTH will seek to explore the cost and benefit trade-offs associated with the use of these products. From a reimbursement perspective, poor or non-existent evidence is not a reason for delaying or avoiding a decision, and so the use of indirect comparison meta-analysis is becoming more common in order to generate the relative efficacy estimates required for a successful health technology assessment in the absence of head to head data.

For all comparisons of dasatinib and nilotinib to imatinib included in the meta-analysis, the derived results are in line with those reported in the actual clinical studies: dasatinib and nilotinib are clinically superior to imatinib in terms of cytogenetic and major molecular response at a range of time points. However, the key value of this paper is in relation to the comparison of dasatinib to nilotinib, since no head to head trial data currently exists. We found no significant difference between the two treatment options in terms of either CCyR or MMR at any of the time points where enough data was available to inform the meta-analysis. Hence, on the basis of the current literature, from the perspective of both clinicians and reimbursement agencies the two products should be viewed as equally efficacious.

To the best of our knowledge, this is the first study to use the combination of a full and thorough systematic review of the literature and subsequent meta-analysis incorporating all available evidence rather than information from single trials (e.g. DASISION and ENESTnd). One other study has been published that attempted to generate relative efficacy estimates for dasatinib and nilotinib via the alteration of the results from one study via a matching algorithm [42]. This approach neglects the body of evidence in other trials – in particular the S0325 Intergroup study [38] – and its acceptability within the reimbursement agencies or with other meta-analysts is untested. Hence, the results from the study presented in this paper are the most robust estimates currently available to both clinicians and reimbursement decision makers.

As noted previously, indirect comparison meta-analysis respects randomisation in all included trials, uses all available evidence (both indirect and head to head) and, assuming the trial populations are broadly homogeneous and endpoints consistently defined, generates results that are consistent with conventional meta-analyses when the amount of head to head evidence is sufficient to allow both approaches to be used. Further, the International Society for Pharmacoeconomics and Outcomes Research (ISPOR) set up a task force to generate ‘best practice’ guidelines to be used in conducting and reporting an indirect comparison meta-analysis. These guidelines have been published and the meta-analysis presented here was conducted to meet the standard laid out in this document [25].

Despite the above mentioned strengths of the analysis, there were a number of limitations. The primary concern is that while the predicted 12 month CCyR values are similar to the values in the ENESTnd study (reported: 80%/78%/65% for nilotinib 600 mg/800 mg and imatinib 400 mg: derived 77.7%/75.7%/62.4% respectively), the predicted value for dasatinib (77.1%) is lower than observed in either of the two identified studies (DASISION: 83.4%, S0325: 82.0%). Given that the *absolute* highest response rates of all included imatinib studies were in these two studies, when the mean baseline imatinib probability and relative *efficacy* estimates from the meta-analysis are combined, the result was inevitable. Hence the studies included may be heterogeneous, although no subgroup specific analyses were done to explore this topic. Further work via statistical comparisons of inclusion criteria, baseline patient characteristics, etc. is required to test this hypothesis although without access to patient level data from multiple pharmaceutical companies such analyses may not be feasible.

Secondary concerns relate to the immaturity of the database and to the limited number of studies identified. This impacted on the project in two ways: i) through the large amount of uncertainty surrounding the derived results and ii) through the inability to generate meaningful evidence networks for other clinically relevant outcome measures (e.g. partial cytogenetic response, progression free survival or overall survival). The majority of these problems will be lessened over time with the publication of either new studies or more information from existing ones. However, given the target population and scarcity of events it may never be possible to differentiate the survival profiles (both progression free and overall) of the two second generation products without a head to head trial.

Thirdly, only one manufacturer (Bristol-Myers Squibb) had the opportunity to provide additional material to the analysis, which could be perceived as non-transparent and a bias in favour of dasatinib. As mentioned in the results section, only material available in the public domain was used in the analysis and also that BMS provided information on Nilotinib related studies. Hence, the review maintained its transparency and no bias existed.

It is important to note that the results were generated using aggregate level trial data and hence should not be interpreted as answering the questions ‘who should get dasatinib?’ and ‘who should get nilotinib?’ Such questions would require either a meta-regression or re-running of the analysis using subgroup specific data.

## Conclusion

Regardless of whether head to head or indirect evidence is considered relevant, treatment for newly diagnosed CML with second generation TKIs such as dasatinib or nilotinib results in significantly higher cytogenetic and molecular response rates compared to imatinib 400 mg daily. However, based on a thorough systematic review of the literature and subsequent meta-analysis there is no statistical difference in the six, 12 and 18 month CCyR rates or the 12 month MMR rates for dasatinib 100 mg daily, nilotinib 600 mg daily and nilotinib 800 mg daily.

## Abbreviations

CCyR: Complete Cytogenic Response; CML: Chronic Myeloid leukemia; CrI: Credible Interval; MMR: Major Molecular Response; NMA: Network Meta-Analysis; QALY: Quality Adjusted Life Year.

## Competing interests

The study was sponsored by a consultancy agreement from Bristol-Myers Squibb Ltd. SM, LB, NH, JC and VE work for an international consultancy firm and have undertaken similar analyses for a number of pharmaceutical firms. No restrictions were, however, placed by Bristol-Myers Squibb ltd. on the design of the study, the choice of included articles or the presentation of results. Publication of the manuscript is not contingent on sponsor approval or censorship of the contents.

## Authors’ contributions

SM, LB, IH and CD designed the study protocol. All authors reviewed manuscripts and undertook data extraction. NH performed the network meta-analysis and all authors contributed to the preparation of the manuscript. All authors read and approved the final manuscript.

## Supplementary Material

Additional file 1 Search Strategies. Click here for file
