# Supplementary material for: The relative efficacy of imatinib, dasatinib and nilotinib for newly diagnosed chronic myeloid leukemia: a systematic review and network meta-analysis
Source: Exp Hematol Oncol. 2013 Feb 19;2:5. doi: 10.1186/2162-3619-2-5 (PMC3585848; doi:10.1186/2162-3619-2-5)
Supplement: Additional file 1 — Search Strategies. [file 2162-3619-2-5-S1.docx]

# Supplementary material

## SEARCH STRATEGIES

The search strategies used to identify randomised studies are presented below

Table S1: Ovid MEDLINE(R) In-Process & Other Non-Indexed Citations and Ovid MEDLINE(R) 1950 to Present

| **#** | **Searches** |
| --- | --- |
| 1 | leukemia/ or leukemia, myeloid/ or expleukemia, myelogenous, chronic, bcr-abl positive/ |
| 2 | (chronicmyel$ adj3 leuk?emia$1).tw. |
| 3 | cml.tw. |
| 4 | leuk?emia$1.tw. |
| 5 | 4 and 3 |
| 6 | 1 or 2 or 5 |
| 7 | (imatinib or gleevec or glivec or sti571 or sti 571 or st 1571 or st1571 or st1 571).mp. |
| 8 | imatinib.nm. |
| 9 | 8 or 7 |
| 10 | (nilotinib or tasigna).mp. |
| 11 | (amn107 or amn 107).mp. [mp=title, original title, abstract, name of substance word, subject heading word, unique identifier] |
| 12 | "4-methyl-N-(3-(4-methylimidazol-1-yl)-5-(trifluoromethyl)phenyl)-3-((4-pyridin-3-ylpyrimidin-2-yl)amino)benzamide)".nm. |
| 13 | 11 or 10 or 12 |
| 14 | (dasatinib or sprycel or bms354825 or bms 354825).mp. |
| 15 | 302962-49-8.rn. |
| 16 | 15 or 14 |
| 17 | exp Interferon-alpha/ |
| 18 | roferon.mp. |
| 19 | intron a.tw. |
| 20 | introna.tw. |
| 21 | 76543-88-9.rn. |
| 22 | 99210-65-8.rn. |
| 23 | (interferon adj1 (alpha$2 or alfa$2)).tw. |
| 24 | ifn a.tw. |
| 25 | 22 or 21 or 18 or 24 or 23 or 19 or 17 or 20 |
| 26 | Hydroxyurea/ |
| 27 | hydroxyurea.tw. |
| 28 | hydroxycarbamid.tw. |
| 29 | 127-07-1.rn. |
| 30 | 27 or 28 or 26 or 29 |
| 31 | 25 or 30 or 16 or 13 or 9 |
| 32 | 6 and 31 |
| 33 | randomized controlled trial.pt. |
| 34 | controlled clinical trial.pt. |
| 35 | randomized controlled trial/ |
| 36 | random allocation.sh. |
| 37 | double blind method.sh. |
| 38 | single blind method.sh. |
| 39 | clinical trial/ |
| 40 | clinical trial/ or clinical trial, phase i/ or clinical trial, phase ii/ or clinical trial, phase iii/ or clinical trial, phase iv/ or multicenter study/ |
| 41 | (clin$ adj25 trial$).ti,ab. |
| 42 | ((singl$ or doubl$ or tripl$ or trebl$) adj25 (blind$ or mask$ or dummy$)).ti,ab. |
| 43 | placebos.sh. |
| 44 | placebo$.ti,ab. |
| 45 | random$.ti,ab. |
| 46 | animals/ not (animals/ and humans/) |
| 47 | or/33-45 |
| 48 | 47 not 46 |
| 49 | 32 and 48 |
| 50 | 49 |
| 51 | limit 50 to english language |
| 52 | 51 |
| 53 | limit 52 to yr="1980 -Current" |

Table S2: EMBASE

| **#** | **Searches** |
| --- | --- |
| 1 | exp *chronic myeloid leukemia/ |
| 2 | (chronic myel$ adj3 leuk?emia$1).tw. |
| 3 | cml.tw. |
| 4 | leuk?emia$1.tw. |
| 5 | 3 and 4 |
| 6 | 1 or 2 or 5 |
| 7 | imatinib/ |
| 8 | (imatinib or gleevec or glivec or sti571 or sti 571 or st 1571 or st1571 or st1 571).mp. |
| 9 | 152459-95-5.rn. |
| 10 | 8 or 7 or 9 |
| 11 | nilotinib/ |
| 12 | 641571-10-0.rn. |
| 13 | (nilotinib or tasigna or amn107 or amn 107).mp. |
| 14 | 11 or 13 or 12 |
| 15 | dasatinib/ |
| 16 | 302962-49-8.rn. |
| 17 | (dasatinib or sprycel or bms354825 or bms 354825).mp. |
| 18 | 16 or 17 or 15 |
| 19 | recombinant alpha2a interferon/ |
| 20 | recombinant alpha2b interferon/ |
| 21 | alpha interferon/ or alpha2a interferon/ or alphan1 interferon/ |
| 22 | 76543-88-9.rn. |
| 23 | 99210-65-8.rn. |
| 24 | 98530-12-2.rn. |
| 25 | roferon.mp. |
| 26 | intron a.mp. |
| 27 | introna.mp. |
| 28 | (interferon adj1 (alpha$2 or alfa$2)).tw. |
| 29 | ifn a.tw. |
| 30 | 27 or 25 or 28 or 21 or 26 or 20 or 22 or 24 or 19 or 23 or 29 |
| 31 | hydroxyurea/ |
| 32 | 127-07-1.rn. |
| 33 | hydroxyurea.tw. |
| 34 | hydroxycarbamid.tw. |
| 35 | 33 or 32 or 34 or 31 |
| 36 | 35 or 18 or 30 or 10 or 14 |
| 37 | 6 and 36 |
| 38 | clinical trial/ |
| 39 | randomized controlled trial/ |
| 40 | randomization/ |
| 41 | crossover procedure/ |
| 42 | double-blind procedure/ |
| 43 | single-blind procedure/ |
| 44 | placebo/ |
| 45 | random$.tw. |
| 46 | rct.tw. |
| 47 | factorial$.tw. |
| 48 | (crossover$ or cross-over$).tw. |
| 49 | placebo$.tw. |
| 50 | (double$ adj blind$).tw. |
| 51 | (singl$ adj blind$).tw. |
| 52 | assign$.tw. |
| 53 | allocat$.tw. |
| 54 | or/38-53 |
| 55 | animal/ not (animal/ and human/) |
| 56 | 54 not 55 |
| 57 | 56 and 37 |
| 58 | 57 |
| 59 | limit 58 to english language |
| 60 | from 59 keep 1-1190 |

## ASSESSMENT OF STUDY QUALITY: Methods and results

The following items were assessed for study quality:

**- Description of randomisation:**

A method of randomisation will be considered adequate when any pattern of allocation is equally likely. Examples of acceptable methods of randomisation include:

- the use of a computer random number generator

- referring to a random number table

The description of randomisation will be regarded as unclear if there is insufficient information about the sequence generation process. For example, a study described as randomised but no method of randomisation is described.

**- Description of allocation concealment:**

An adequate method of allocation concealment ensures patients and investigators are unaware of the forthcoming assignment. Examples of appropriate methods of allocation concealment include the following:

- central allocation: telephone, pharmacy-controlled

- sequentially numbered, opaque, sealed envelopes

- sequentially numbered drug containers of identical appearance

Inadequate methods of allocation concealment include:

- the use of open random allocation schedule: list of random numbers
- use of non-opaque or not sequentially numbered envelopes
- allocation by alternation, date of birth

If there is insufficient information to assess the method of allocation concealment this will be regarded as unclear.

- **Detailed inclusion/exclusion criteria**

An adequate description of inclusion/exclusion criteria will include a comprehensive description of the eligibility criteria used to select the trial participants.

**- Blinding of participants, investigators and outcome assessors:**

Criteria for an adequate method of blinding will be based on the following:

- blinding of patients and investigators is ensured for example a statement indicating the use of “identical placebo” or “double-dummy”.
- Either patients or investigators were not blinded but the outcome assessment was blinded

The following will be regarded as inadequate method of blinding:

- no blinding or incomplete blinding
- study described as double blind but likely that blinding could have been broken

Method of blinding will be regarded as unclear if there is insufficient information to assess blinding. For example, a study described as double-blind but no description of blinding is provided.

**- Description of patients’ baseline characteristics- whether the groups were similar at the outset of the study in terms of prognostic factors:**

We will assess the reporting of patient’s baseline characteristics. Adequate reporting should include a detailed description of the main prognostic factors of patients at baseline per treatment group and report any unbalances between the groups.

Inadequate description of patient’s baseline characteristics will include: no description or insufficient information of patient’s baseline characteristics or details provided for the whole group of patients in the study rather than per treatment arms.

**-Intention-to-treat analysis**

Very often, patients enrolled in a randomised trial do not receive the study treatment to which they were randomised or are withdrawn from the study. An intention-to-treat (ITT) analysis includes data on all trial patients and analyses them according to the intervention to which they were randomised.

While it is possible to analyse data of patients according to the intervention groups they were allocated to, it is not always possible to measure outcome data on all the patients due to withdrawals and drop-outs. For this reason a true ITT analysis cannot be performed without making assumptions about missing outcome data.

It is common for authors to report an ITT analysis even though some outcome data are missing.

An ITT analysis will be regarded as adequate if there is a statement confirming that the analysis was based on ITT or if it is clear from the text that data from patients were analysed according to the intervention group they were randomised. As regards whether appropriate methods were used to account for missing data, we will highlight where there is a high proportion of missing outcomes or large difference between groups.
